# Supplementary material for: Climate change influences on the geographic distributional potential of the spotted fever vectors Amblyomma maculatum and Dermacentor andersoni
Source: PeerJ. 2022 May 3;10:e13279. doi: 10.7717/peerj.13279 (PMC9074859; doi:10.7717/peerj.13279)
Supplement: Supplemental Information 2 [file peerj-10-13279-s002.docx]

**Supplementary Materials**

**S1 file.** Occurrence points used in this study.

**Table S2.** Best models selected based on parameter settings in the process of model calibration, to produce final models for *A. maculatum and D. andersoni*. Reg. = regularization; AUC = area under the curve; ROC = receiver operating characteristic; AICc = Akaike information criterion corrected for small sample size. Best sets of environmental combinations were set 1 (PC1-PC4), set 2 (PC1-PC3), set 3 (PC1, PC2, PC4), set 4 (PC1, PC3, PC4), set 6 (PC1, PC2), and set 7 (PC1, PC3).

| Feature class | Reg. multiplier | Variable set | Mean AUC ratio | partial ROC | Omission rate at 5% | AICc | Delta AICc | Parameters |
| --- | --- | --- | --- | --- | --- | --- | --- | --- |
| ***Amblyomma maculatum*** | | | | | | | | |
| L, Q | 0.1 | Set 3 | 1.35 | 0 | 0.022 | 1738.395 | 0 | 6 |
| L, Q | 0.2 | Set 3 | 1.35 | 0 | 0.022 | 1738.417 | 0.022 | 6 |
| L, Q | 0.3 | Set 3 | 1.35 | 0 | 0.022 | 1738.443 | 0.047 | 6 |
| L, Q | 0.1 | Set 6 | 1.35 | 0 | 0.022 | 1735.467 | 0.071 | 4 |
| L, Q | 0.4 | Set 3 | 1.35 | 0 | 0.022 | 1738.473 | 0.077 | 6 |
| L, Q | 0.2 | Set 6 | 1.35 | 0 | 0.022 | 1738.478 | 0.082 | 4 |
| L, Q | 0.3 | Set 6 | 1.36 | 0 | 0.022 | 1738.49 | 0.095 | 4 |
| L, Q | 0.5 | Set 3 | 1.35 | 0 | 0.022 | 1738.504 | 0.108 | 6 |
| L, Q | 0.4 | Set 6 | 1.35 | 0 | 0.022 | 1738.505 | 0.109 | 4 |
| L, Q | 0.5 | Set 6 | 1.35 | 0 | 0.022 | 1738.52 | 0.125 | 4 |
| L, Q | 0.6 | Set 6 | 1.35 | 0 | 0.22 | 1738.537 | 0.142 | 4 |
| L, Q | 0.6 | Set 3 | 1.35 | 0 | 0 | 1738.53 | 0.143 | 6 |
| L, Q | 0.7 | Set 6 | 1.35 | 0 | 0.022 | 1738.556 | 0.160 | 4 |
| L, Q | 0.7 | Set 3 | 1.35 | 0 | 0 | 1738.576 | 0.180 | 6 |
| L, Q | 0.8 | Set 6 | 1.34 | 0 | 0.022 | 1738.576 | 0.180 | 4 |
| L, Q | 0.9 | Set 6 | 1.35 | 0 | 0.022 | 1738.598 | 0.202 | 4 |
| L, Q | 0.8 | Set 3 | 1.35 | 0 | 0 | 1738.618 | 0.222 | 6 |
| L, Q | 1 | Set 6 | 1.35 | 0 | 0.022 | 1738.62 | 0.225 | 4 |
| L, Q | 0.9 | Set 3 | 1.35 | 0 | 0 | 1783.661 | 0.265 | 6 |
| L, Q | 1 | Set 3 | 1.35 | 0 | 0 | 1738.707 | 0.311 | 6 |
| L, Q | 2 | Set 6 | 1.35 | 0 | 0 | 1738.925 | 0.529 | 4 |
| L, Q, P | 2 | Set 6 | 1.37 | 0 | 0.022 | 1739.225 | 0.830 | 4 |
| L, Q | 2 | Set 3 | 1.36 | 0 | 0 | 1739.322 | 0.927 | 6 |
| L, Q, H | 2 | Set 3 | 1.36 | 0 | 0 | 1739.324 | 0.928 | 6 |
| L, Q | 3 | Set 6 | 1.36 | 0 | 0 | 1739.359 | 0.963 | 4 |
| L, Q, H | 3 | Set 6 | 1.37 | 0 | 0 | 1739.359 | 0.963 | 4 |
| L, Q | 0.1 | Set 2 | 1.37 | 0 | 0.022 | 1739.771 | 1.376 | 6 |
| L, Q | 0.1 | Set 1 | 1.36 | 0 | 0.022 | 1739.786 | 1.390 | 8 |
| L, Q | 0.2 | Set 2 | 1.37 | 0 | 0.022 | 1739.788 | 1.392 | 6 |
| L, Q | 0.3 | Set 2 | 1.36 | 0 | 0.022 | 1739.808 | 1.413 | 6 |
| L, Q | 0.2 | Set 1 | 1.37 | 0 | 0.022 | 1739.81 | 1.415 | 8 |
| L, Q | 0.4 | Set 2 | 1.37 | 0 | 0.022 | 1739.832 | 1.437 | 6 |
| L, Q | 0.3 | Set 1 | 1.36 | 0 | 0.022 | 1739.839 | 1.44 | 8 |
| L, Q | 0.5 | Set 2 | 1.37 | 0 | 0.022 | 1739.859 | 1.46 | 6 |
| L, Q | 0.4 | Set 1 | 1.36 | 0 | 0.022 | 1739.876 | 1.48 | 8 |
| L, Q | 0.6 | Set 2 | 1.37 | 0 | 0.022 | 1739.891 | 1.49 | 6 |
| L, Q, H | 4 | Set 6 | 1.37 | 0 | 0 | 1739.912 | 1.51 | 4 |
| L, Q | 4 | Set 6 | 1.37 | 0 | 0 | 1739.913 | 1.51 | 4 |
| L, Q | 0.5 | Set 1 | 1.36 | 0 | 0.022 | 1739.914 | 1.51 | 8 |
| L, Q | 3 | Set 1 | 1.38 | 0 | 0.022 | 1739.92 | 1.52 | 7 |
| L, Q, H | 3 | Set 1 | 1.38 | 0 | 0.022 | 1739.925 | 1.52 | 7 |
| L, Q | 0.7 | Set 2 | 1.37 | 0 | 0.022 | 1739.928 | 1.53 | 6 |
| L, Q | 0.6 | Set 1 | 1.36 | 0 | 0.022 | 1739.961 | 1.56 | 8 |
| L, Q | 0.8 | Set 2 | 1.36 | 0 | 0.022 | 1739.967 | 1.57 | 6 |
| L, Q, P | 3 | Set 6 | 1.37 | 0 | 0.022 | 1739.967 | 1.57 | 4 |
| L, Q, P, H | 3 | Set 6 | 1.38 | 0 | 0.022 | 1739.967 | 1.57 | 4 |
| L, Q | 0.9 | Set 2 | 1.37 | 0 | 0.022 | 1740.009 | 1.61 | 6 |
| L, Q | 0.7 | Set 1 | 1.37 | 0 | 0.022 | 1740.012 | 1.616 | 8 |
| L, Q | 1 | Set 2 | 1.36 | 0 | 0.022 | 1740.053 | 1.658 | 6 |
| L, Q | 0.8 | Set 1 | 1.37 | 0 | 0.022 | 1740.068 | 1.67 | 8 |
| L, Q | 0.9 | Set 1 | 1.36 | 0 | 0.022 | 1740.128 | 1.733 | 8 |
| L, Q, H | 3 | Set 3 | 1.36 | 0 | 0 | 1740.191 | 1.795 | 6 |
| L, Q | 3 | Set 3 | 1.36 | 0 | 0 | 1740.192 | 1.796 | 6 |
| L, Q | 1 | Set 1 | 1.36 | 0 | 0.022 | 1740.195 | 1.800 | 8 |
| L, Q, H | 0.9 | Set 6 | 1.36 | 0 | 0.022 | 1740.289 | 1.893 | 9 |
| ***Dermacentor andersoni*** | | | | | | | | |
| L, Q, H | 1 | Set 7 | 1.24 | 0 | 0.04 | 1592.484 | 0 | 5 |
| L, Q, P | 2 | Set 4 | 1.23 | 0 | 0.04 | 1593.605 | 1.12 | 7 |

**Figures**

**Fig. S3.**

**
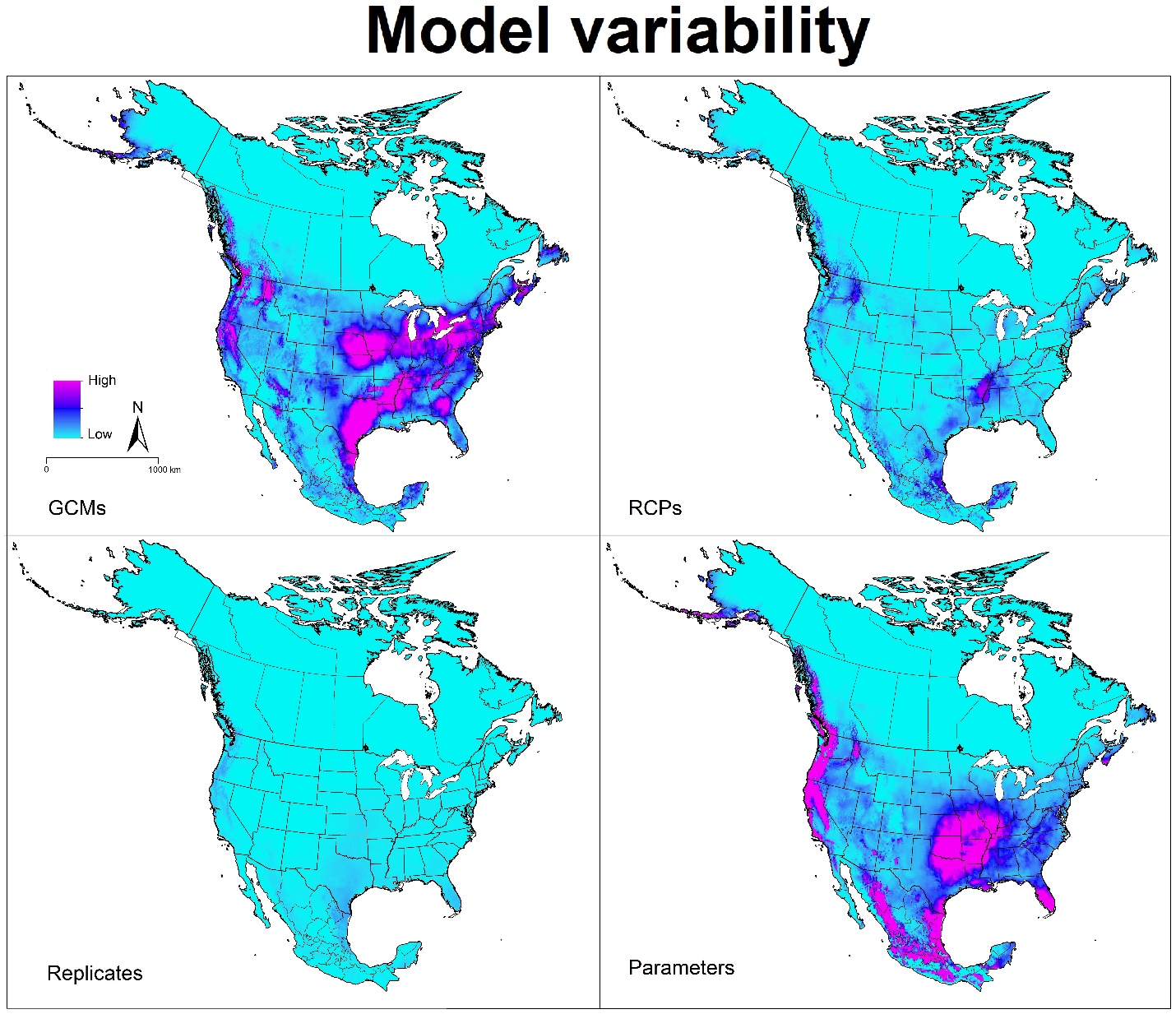
**

*Amblyomma maculatum*: Median of variance coming from replicates, parameters settings, general circulation models (GCMs) and representative concentration pathway scenarios (RCPs) in future projections.

**Fig. S4.**

**
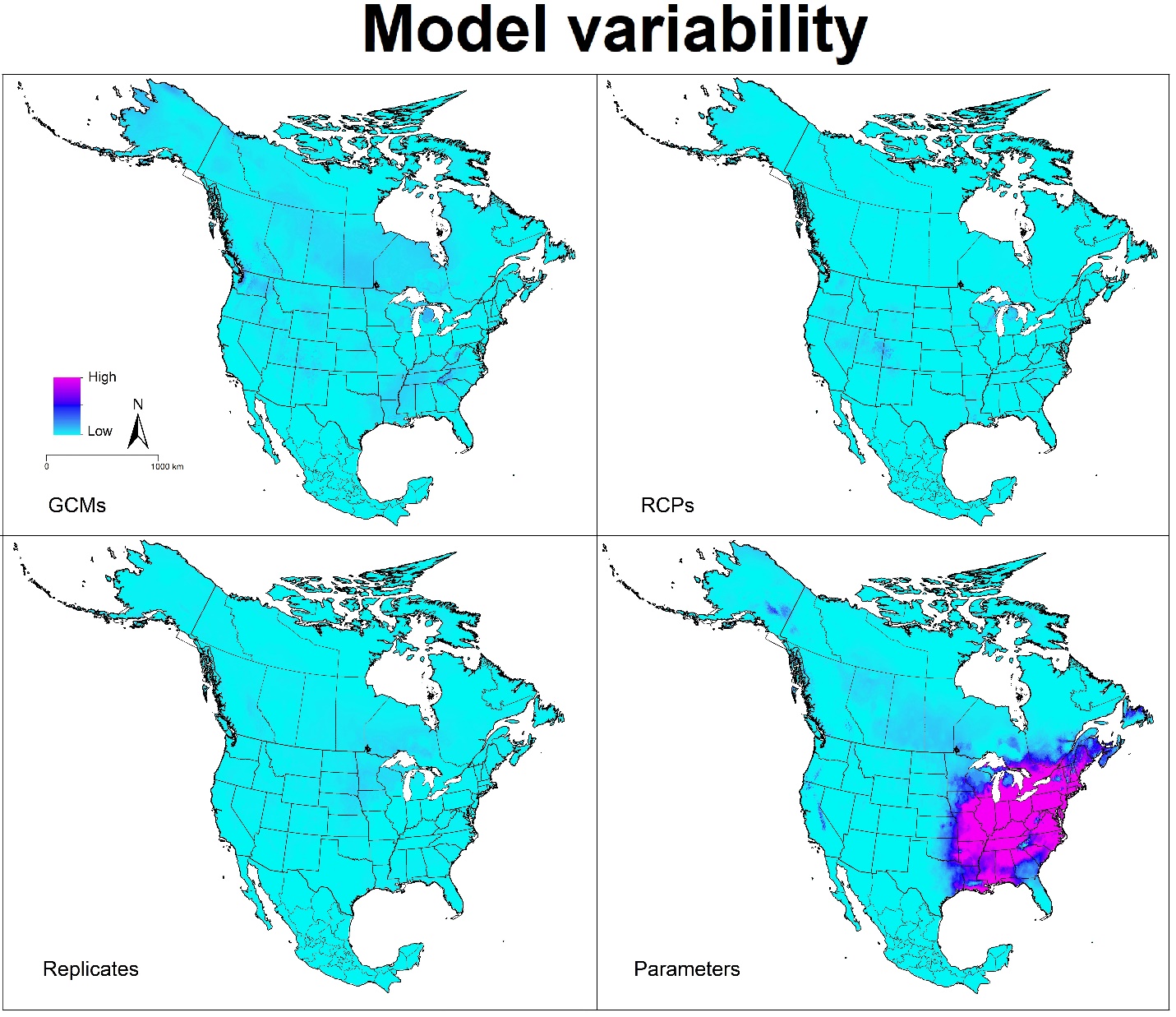
**

*Dermacentor andersoni*: Median of variance coming from replicates, parameters settings, general circulation models (GCMs) and representative concentration pathway scenarios (RCPs) in future projections.
